# Polysaccharide-Based Hydrogels and Their Application as Drug Delivery Systems in Cancer Treatment: A Review

**DOI:** 10.3390/jfb14020055

**Published:** 2023-01-19

**Authors:** Marco Dattilo, Francesco Patitucci, Sabrina Prete, Ortensia Ilaria Parisi, Francesco Puoci

**Affiliations:** 1 Department of Pharmacy, Health and Nutritional Sciences, University of Calabria, 87036 Rende, CS, Italy; 2 Macrofarm s.r.l., c/o Department of Pharmacy, Health and Nutritional Sciences, University of Calabria, 87036 Rende, CS, Italy

**Keywords:** polysaccharide, hydrogels, cancer treatment, drug delivery, chitosan, alginate, hyaluronic acid, cellulose, carrageenan

## Abstract

Hydrogels are three-dimensional crosslinked structures with physicochemical properties similar to the extracellular matrix (ECM). By changing the hydrogel’s material type, crosslinking, molecular weight, chemical surface, and functionalization, it is possible to mimic the mechanical properties of native tissues. Hydrogels are currently used in the biomedical and pharmaceutical fields for drug delivery systems, wound dressings, tissue engineering, and contact lenses. Lately, research has been focused on hydrogels from natural sources. Polysaccharides have drawn attention in recent years as a promising material for biological applications, due to their biocompatibility, biodegradability, non-toxicity, and excellent mechanical properties. Polysaccharide-based hydrogels can be used as drug delivery systems for the efficient release of various types of cancer therapeutics, enhancing the therapeutic efficacy and minimizing potential side effects. This review summarizes hydrogels’ classification, properties, and synthesis methods. Furthermore, it also covers several important natural polysaccharides (chitosan, alginate, hyaluronic acid, cellulose, and carrageenan) widely used as hydrogels for drug delivery and, in particular, their application in cancer treatment.

## 1. Introduction

Hydrogels are three-dimensional, insoluble, crosslinked polymer networks that can hold large amounts of water and biological fluids in their swollen state. Due to their significant water content, hydrogels have a degree of elasticity that is close to that of natural tissue, making biocompatibility one of their most impressive features [1]. Hydrogels are considered smart biomedical materials able to respond the fluctuations in environmental stimuli, including pH, temperature, magnetic and electric fields, and ionic strength [2]. Polymers’ properties and the density of crosslinking influence the sensitivity of hydrogels to external stimuli. Over the years, hydrogels have been investigated for a wide range of applications, from biomedical and industrial to agricultural and environmental areas. The growing availability of functional monomers and crosslinking agents is increasing the spectrum of their applicability. Because of their porous characteristics, hydrogels are ideal materials for drug loading and delivery [3]. They can adsorb and store many types of medicines, allowing their release at predetermined rates for certain periods of time. Hydrogels can function as a natural extracellular matrix (ECM), promoting cell proliferation and tissue regeneration [4]. They also find application in regenerative medicine, promoting tissue regrowth and bone repair work [5]. In recent years, several hydrogel-based systems have been developed for cancer prevention, diagnosis, and treatment. As a matter of fact, the low toxicity, biocompatibility, and biodegradability make this material a promising anticancer system, able to promote the localized, sustained, and controlled release of chemotherapeutics, increase the therapeutic index of the drugs, and reduce the consequent side effects. Many hydrogel formulations have received European Medicines Agency (EMA) and/or Food and Drug Administration (FDA) approval as drug carrier materials and have been promoted to clinical practice [6]. Currently, an increasing number of immunotherapeutic agents, ranging from small-molecule drugs to macromolecular drugs, have been explored and incorporated into hydrogel-based systems via various physical or chemical mechanisms for enhanced cancer immunotherapy with reduced side effects [7].

The aim of this review is to describe the state of art of different natural hydrogels based on polysaccharides and discuss their application as drug delivery systems with a focus on cancer treatment.

## 2. Materials and Methods

A review of the literature was performed using PubMed and Scopus as our main search engines. Results were then filtered for full-text English-language primary research articles published in the field of polysaccharide-based hydrogel and drug delivery systems from 2013 up to 2023, using terms “hydrogel” AND “polysaccharide” AND “Drug delivery system” AND “Cancer treatment”. The search formula was developed in accordance with the characteristics of different databases. All the information extracted from selected papers was grouped and the review was organized focusing on the following main items: hydrogel classification, properties and synthesis methods, natural hydrogels in drug delivery systems (chitosan, sodium alginate, hyaluronic acid, cellulose, carrageenan), and natural hydrogels in cancer treatment.

## 3. Classification

**Figure 1:**
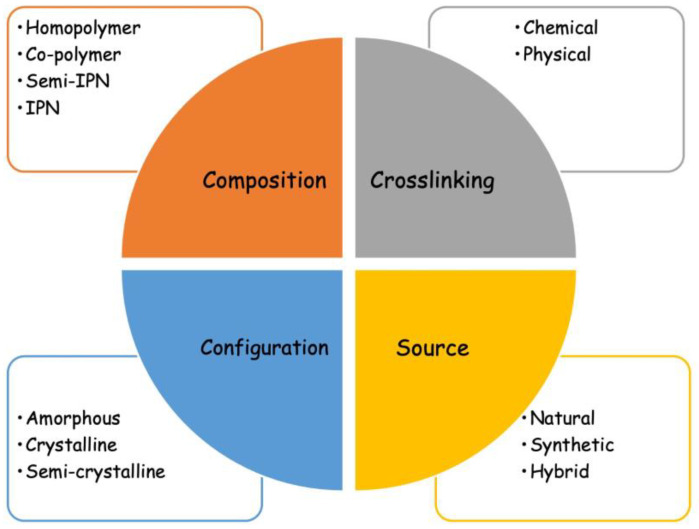
Classification of hydrogels.

A number of classifications of hydrogels are reported in the literature, such as the type of crosslinking, polymeric composition, source of polymeric materials, and configuration (Figure 1).

### 3.1. Crosslinking

Hydrogels can be held together by chemical or physical interactions: they are called “chemical” or “permanent” when they are covalently crosslinked networks and they are called “physical” or “reversible” when the crosslinking is formed by chain entanglements and/or hydrogen bonds or hydrophobic or ionic interactions [8]. Chemical hydrogels are usually prepared by two different methods: “three-dimensional polymerization” in the presence of a hydrophilic monomer and crosslinking agent, or by the direct crosslinking of water-soluble polymers. The reaction is usually initiated by the addition of a free radical initiator or by irradiation using UV/gamma-rays or electron beams [9]. The three-dimensional polymerization usually results in new materials carrying a large amount of unreacted monomers, which can often be toxic. Therefore, a purification step is required. To avoid the need for purification, an additional post-polymerization process can be added, leading to a higher degree of monomer conversion. Moreover, the use of non-toxic monomers represents an alternative method [10]. Another method for hydrogel synthesis is the crosslinking of water-soluble polymers. These materials are non-toxic and already used in several biomedical applications. Both the radiation and thermal crosslinking of water-soluble polymers do not need any purification step and allow the formation of a hydrogel and its sterilization [2]. Physical hydrogels have weaker connections than chemically crosslinked hydrogels. Nevertheless, their popularity is increasing since they are easy to prepare and do not require the use of organic solvents or of a crosslinking agent [11].

### 3.2. Polymeric Composition

There is also a classification of hydrogels based on their polymeric composition. Homopolymeric hydrogels are referred to as polymer networks derived from a single species of monomer [1]. They can be crosslinked or uncrosslinked according to the nature of the monomer and the polymerization technique. Crosslinked homopolymers are particularly used in drug carriers and in contact lenses [12]. Copolymeric hydrogels consist of two or more different monomers with at least one hydrophilic component, organized in any configuration, such as random, block, or alternating along with the polymer chain [13]. An interpenetrating network is composed of two independent crosslinked polymeric chains, which may be natural or synthetic, and one of them is synthesized or crosslinked in the immediate presence of the other. In a semi-IPN, only one component is crosslinked, and the other component is a non-crosslinked polymer [14].

### 3.3. Source

According to their composition, hydrogels can be divided into natural, synthetic, or hybrid. Synthetic hydrogels can be designed to have specific mechanical and chemical properties according to the application of the material, and they are still in continuous developing stages. Hybrid hydrogels are a combination of natural and synthetic polymers [15]. In the literature, different combinations of proteins/polysaccharides with synthetic monomers to obtain hybrid hydrogels are reported. Natural hydrogels have strong cell adhesion properties and are biocompatible and biodegradable. The two major classes of natural polymers used for this type of hydrogel are polysaccharides and proteins. Natural hydrogels have several advantages over synthetic ones, such as their inherent biocompatibility, biodegradability, and safety. Moreover, they are readily available from renewable resources, such as animals, plants, algae, and the fermentation of microorganisms [16].

### 3.4. Other Classifications of Hydrogels

The physical structure and chemical composition allow us to distinguish hydrogels as amorphous, crystalline, and semicrystalline (a complex mixture of amorphous and crystalline phases). According to the technique of polymerization involved in the synthesis, hydrogels can appear as a matrix, film, or microsphere. Based on the presence or absence of an electrical charge located on the crosslinked chains, hydrogels may be classified as nonionic (neutral); ionic; amphoteric electrolytes containing both acidic and basic groups; or zwitterionic containing both anionic and cationic groups in each structural repeating unit [3]. The main criteria for hydrogels’ classification and their features are summarized in Table 1.

## 4. Properties

In recent years, the preparation of hydrogels has become a matter of great interest. Hydrogels possess high water content, biocompatibility, and chemical and physical properties that make them reliable for application in the biomedical and commercial industries [17].

### 4.1. Swelling Properties

Swelling is the characteristic property of hydrogels, which depends on several environmental factors, such as pH, temperature, ionic strength, and crosslinking ratio. Hydrogels are made up of polymers that are crosslinked to one another. When the polymer chain interacts with the solvent, it starts growing to the fully expanded state [18]. The crosslinking ratio affects the swelling behavior, applying an opposite force. Therefore, highly crosslinked hydrogels will have a lower swelling ratio and vice versa. The equilibrium state between these two forces illustrates the swelling behavior of hydrogels (Equation (1)):

Equilibrium swelling ratio = W_Swollen_/W_Dry_ (1)

where W_Swollen_ represents the weight of the swollen hydrogel and W_Dry_ represents the weight of the dry hydrogel [19].

The swelling degree is the main parameter that can be evaluated on hydrogel samples to discriminate between the crosslinked gel and the non-crosslinked original material. It is a simple and quick test that requires small amounts of sample, minimal technical skill, and inexpensive equipment [20].

### 4.2. Mechanical Properties

Hydrogels’ mechanical properties are very important for pharmaceutical and biomedical applications such as drug delivery matrices, wound dressing materials, ligament and tendon repair, and tissue engineering. A hydrogel is a tailor-made material that can be mechanically optimized by changing its crosslinking ratio. The higher the degree of crosslinking is, the stronger the hydrogel will be, even though the percentage of elongation will decrease, creating a more brittle structure. Through the choice of polymer network or degree of crosslinking, it is then possible to adjust the mechanical properties of hydrogels to meet the requirements of the final application [21]. Hybridization with other polymers, nanoparticles, and nanofibers has been used to improve the mechanical strength and make hydrogels more stable and able to bear loads and fulfill their purpose [22]. Moreover, matrix stiffness has been reported to affect cell activities, by interfering with cells’ spreading, migration, proliferation, and differentiation [23].

### 4.3. Biocompatibility and Biodegradability

Hydrogels have attracted a lot of interest in the pharmaceutical and biomedical fields because of their biocompatible and biodegradation properties. Biocompatibility is a term used to describe the ability of a material to exist in harmony with surrounding tissues without causing a host reaction. To be considered biocompatible, a material must pass cytotoxicity and in vivo toxicity studies [24]. Biocompatibility consists of two components: biosafety and biofunctionality. The former represents a lack of cytotoxicity, mutagenesis, or carcinogenesis, while the latter is the ability of a substance to achieve the task for which it was planned [18]. Hydrogels need to be biocompatible and non-toxic. In the case of natural hydrogels, most polysaccharides and proteins are found to be biocompatible and functional as biomaterials. For synthetic hydrogels, many chemicals used in the polymerization may represent an issue for the biocompatibility of the material. Unreacted monomers, initiators, surfactants, or organic solvents used in hydrogel synthesis may also constitute a challenge. Therefore, these materials need to be purified of remaining dangerous chemicals before use [25].

Hydrogels used for biomedical application are often biodegradable. Biodegradability can be defined as the conversion of materials into less complex products by solubilization, hydrolysis, or the action of biological substances. Based on these mechanisms, it is possible to design hydrogels with good biodegradation properties and preferable degradation rates. Biodegradable systems have been used quite broadly in the drug delivery area since they can degrade and be cleared from the body, eliminating the need for removing the delivery system after the entirety of the drug has been released [24].

## 5. Natural Hydrogels

Natural hydrogels, which this review will focus on, have drawn attention due to their unique properties: biocompatibility, biodegradability, low cytotoxicity, as well as their similarity to the physiological environment and their support of cell adhesion and proliferation [26]. Hyaluronic acid (HA), alginate (ALG), and collagen are some commonly used natural polymers in pharmacy, biomedical areas, and cancer treatment. However, most natural polymers do not have strong mechanical properties and do not always fulfil clinical requirements [27]. There are three major classes of natural polymers:

- Polysaccharides, such as chitosan (CHI), HA, and ALG;
- Proteins, such as gelatin, collagen and silk, or peptides [28];
- DNA.

The combination of polysaccharides and proteins has displayed several advantages over the use of either material alone [29]. Segura et al. developed a substrate-mediated approach to deliver DNA from HA–collagen hydrogels [30]. DNA complexes were immobilized on the HA–collagen substrate and then released by hyaluronidase treatment, which degraded the HA backbone. The biodegradable scaffold was able to transfect adherent cells using the immobilized DNA, preventing its distribution to non-target tissues. Kim et al. prepared a CHI/gelatin scaffold as an in situ injectable hydrogel based on the improved mechanical and biological performance provided by the two natural polymers [31]. An acid-tolerant tyrosinase, tyrosinase-CNK, catalyzed the reaction between chitosan and gelatin, forming a permanent hydrogel. The synthesized matrix showed exceptionally high swelling and in vitro degradability properties, representing a suitable biomaterial for tissue engineering and drug delivery.

Natural polymers have been studied for different applications, including drug delivery for the treatment of cancer and infections, wound healing, tissue engineering, and regenerative medicine. The unique physical and chemical properties of hydrogels have drawn particular attention regarding their use in drug delivery applications [32].

Drug delivery approaches require some general guidelines in order to obtain a safe transport system able to achieve the desired therapeutic outcome and to avoid side effects. The functionalization with drugs and other therapeutic agents can be performed with two different methods: either by mixing the drug with the other reagents before the beginning of the reaction, or once the hydrogel is achieved. The first one focuses on the dissolution of the drug into the water and is more suitable for hydrophilic drugs, while the second one refers to dry hydrogels soaked in a standard drug solution and is called post-loading [33]. Appropriate procedures have been validated to quantify the released drug from hydrogels, using practical optical spectroscopy methods or more sensitive high-performance liquid chromatography (HPLC), mass spectrometry, or polymerase chain reaction (PCR) [34]. To date, numerous hydrogel systems have been used to increase the efficacy and reduce the cytotoxicity of potent drugs in the treatment of several diseases.

### 5.1. Synthesis

**Figure 2:**
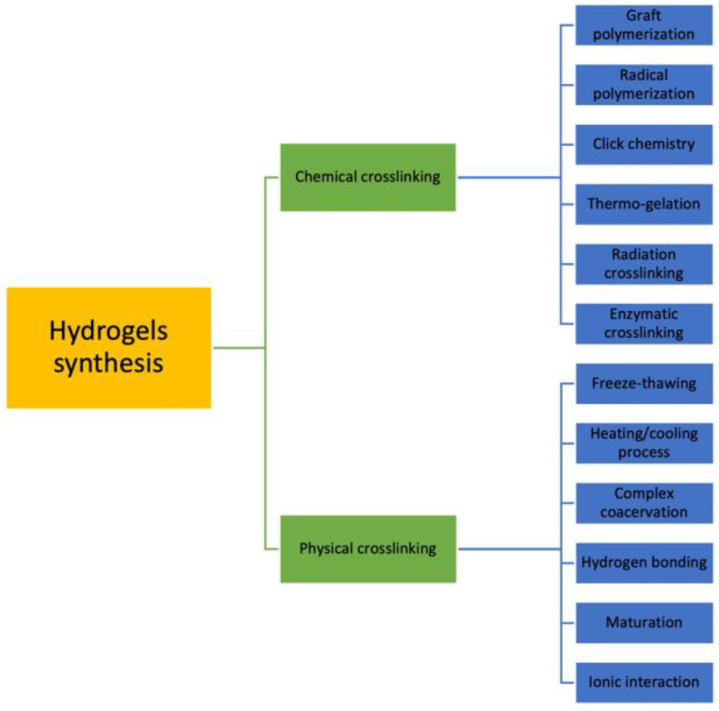
Different methods for hydrogel preparation.

Hydrogels can be synthesized by various techniques, usually divided into two main groups: chemical crosslinking and physical crosslinking (Figure 2). As mentioned before, chemically crosslinked hydrogels are characterized by permanent junctions, while physically crosslinked hydrogels have reversible bonds that result from polymer chain entanglements or physical interactions. Chemical methods include grafting, radical polymerization, click chemistry, thermo-gelation, and radiation crosslinking [35].

However, natural hydrogels are mostly synthesized by self-assembly physical crosslinking processes, such as ionic interactions, hydrogen bonds, or hydrophobic interactions. The temperature, electric field, magnetic field, light, or pressure are responsible for polymers’ phase transition and the formation of reversible connections. Physically crosslinked hydrogels can be unstable and undergo fast decomposition, limiting their applications. Nevertheless, in the past ten years, they have become increasingly popular because of the easier synthesis process [36]. Some methods reported in the literature to obtain physically cross-linked hydrogels are as follows.

#### 5.1.1. Ionic Interaction

The addition of counter-ions to a polymer solution can induce gelation through ionic bond formation [5]. This reaction, called ionic gelation, allows us to obtain nanoparticles with a regular surface due to the electrostatic interactions between polyelectrolytes and the counter-ions. Ionic gelation is typically used for the preparation of ALG hydrogels: ALG’s anionic groups can react with divalent cations to form insoluble networks. Salts of calcium or zinc are usually used for crosslinking with ALG [37]. CHI’s cationic groups’ reaction with the polyanion of sodium triphosphate (TPP) also represents an ionic gelation approach: when TPP is dissolved into a CHI solution, the polyanion binds to a positive amino group by electrostatic interaction. This represents the starting point of the gelation process that will lead to the formation of CHI nanoparticles [38].

#### 5.1.2. Freeze-Drying

Cryogelation is one of the newest methods to develop polysaccharide-based hydrogels for biomedical purposes. It consists of the solubilization of the polymer in an appropriate solvent, followed by regeneration (solvent removal) and freezing with the formation of ice crystals. The freeze-drying process will generate pores and a complex three-dimensional structure with similar properties to natural soft tissues. The origin of the polymer, dissolution parameters, and type of solvent, as well as the temperature and freezing rate, can affect the formation of cryogels [39].

#### 5.1.3. Heating/Cooling Process

Physically crosslinked hydrogels can be synthesized by heating and then cooling a polymer solution. This is the case for carrageenan (CRG): in an aqueous medium, CRG molecules are in the random coil form at a sufficiently high temperature and are then converted into a double-helical conformation by cooling down the solution. This gelation process is called the “sol–gel transition” and can be reversed by an increase in the temperature [40]. Recently, Deng et al. developed an adenine-modified CHI hydrogel by a simple heating/cooling process with excellent self-healing, biocompatible, and antibacterial properties and cell proliferation activity for potential application in wound healing (Deng, 2022 #173).

#### 5.1.4. Complex Coacervation

Polyelectrolytes bearing ionizable groups that can dissociate in an aqueous medium can establish electrostatic interactions to form new materials. Complex coacervations occur between oppositely charged polymers that stick together, forming soluble and insoluble complexes according to the pH of the different solutions. An example is the reaction between HA, a weak polyanion, and CHI, a cationic polysaccharide [41]. In recent years, protein/polysaccharide complexes have been widely used in novel food processing applications and as biopolymers for loading functional components (Li, 2021 #175). pH-induced coacervates between gliadin nanoparticles and ALG have been developed for the encapsulation and controlled release of curcumin. The complex remained stable at a wide range of pH values and provided a protective effect for curcumin, achieving sustained and controlled release (Su, 2021 #174).

#### 5.1.5. Hydrogen Bonding

Hydrogen bonding between polymeric chains can lead to the formation of a hydrogel through the association of an electron-deficient hydrogen atom and a highly electronegative functional group [42]. However, this method is very sensitive to different parameters, such as the degree of polymer functionality, the concentration of the solution, or the temperature [43]. Ahmadian et al. realized a gelatin–tannic acid hydrogel by means of hydrogen bonding between the hydroxyl groups of tannic acid as hydrogen donors and the carboxyl and amine groups of gelatin as hydrogen acceptors. It represents a simple, safe, cheap, and scalable approach that does not require an additional chemical crosslinker. This system showed considerable mechanical properties and enhanced therapeutic effects on wound repair in vivo in a skin defect model (Ahmadian, 2021 #176).

Physical crosslinking can also take place by lowering the pH of a polymer solution, promoting hydrogen bond formation. Carboxymethylcellulose (CMC), for example, can be crosslinked by immersion in acid solution. The gelation process takes place via the diffusion of the acid into the polymer chains and the substitution of the sodium in CMC with hydrogen. Hydrogen bonds are formed between molecules and a decrease in CMC solubility is observed [44].

### 5.2. Polysaccharides

Among the biopolymers used in the preparation of hydrogels, polysaccharides have several advantages that make them the polymer group with the longest-standing as well as the widest-ranging experience in the biomedical field [45]. Polysaccharides have a number of advantages compared to synthetic polymers: they are easily accessible, biocompatible and biodegradable, and non-toxic, since the monomer residues are not dangerous to health and have high water solubility. Moreover, because of the ease of their production, several polysaccharides are cheaper than synthetic polymers [46]. Table 2 reports a summary of recently used polysaccharide-based hydrogels as drug delivery systems.

#### 5.2.1. Chitosan

Chitosan (CHI) is one of the most common materials used in hydrogel synthesis. It is obtained by the deacetylation of chitin extracted from the endoskeletons of marine crustaceans. Chitin has a rigid crystalline structure and low solubility in water due to the presence of intra-chain hydrogen bonds [61]. The charge density of this polymer is correlated to the degree of deacetylation, which is the percentage of glucosamine monomers present in the chitin structure. Its high level of acetylated groups means that chitin is not easy to use. This parameter plays an important role in the solubility of chitin, which is insoluble in water containing acetic acid [62]. As a matter of fact, when the degree of deacetylation reaches around 50%, chitin is converted to CHI and becomes soluble in aqueous acidic media. When chitin is deacetylated to CHI, the number of amino groups increases, leading to a positively charged polymer [63]. CHI is a polysaccharide consisting of two sub-units, D-glucosamine and N-acetyl-D-glucosamine linked by a *β* (1 → 4) glycosidic bond. It has been used for biomedical and cosmetic applications because of its many properties, such as biocompatibility, biodegradability, and non-toxicity [64]. Moreover, CHI presents antibacterial activity, related to the presence of amino groups in its backbone that promote binding to the negatively charged bacterial cell walls, resulting in the alteration of the cell envelope structures and permeability [65]. However, its high molecular weight and low solubility in aqueous media due to its crystalline structure limit the use of CHI in the food industry or in the medical field. Its cationic character makes it unique among all the other biopolymers and a perfect candidate to produce hydrogels for drug delivery applications [66]. When dissolved in an acidic solution, CHI amines protonate and coagulate upon the addition of macromolecules having an anionic nature, producing a possible delivery system for the sustained release of therapeutics [67]. Bhise et al. explored the ionic interactions of Naproxen sodium and CHI, confirming the complex formation between ionic drugs and oppositely charged polyelectrolytes. Moreover, the addition of a release-controlling external polymer, hydroxypropylmethylcellulose (HPMC), increased the matrix integrity and allowed for more controlled and retarded drug release [68]. CHI’s low solubility at a neutral pH is an important limitation, but the combination with other polymers or inorganic materials may help in overcoming this drawback, maintaining the structural integrity of the biomaterials. Cheng et al., for instance, developed a thermosensitive hydrogel combining CHI with gelatin and using *β*-glycerophosphate as a crosslinker for adipose-derived stem cells’ (ASCs) sustained release. In comparison to pure CHI hydrogels, CHI/gelatin hydrogels were able to release a higher number of encapsulated cells. Moreover, after blending gelatin in the CHI hydrogel, the cell survival significantly improved because of the alteration in the surface charge and the increased hydrophilicity [69]. Recently, CHI nanohydrogels have been synthesized through covalent and non-covalent interactions, using formaldehyde crosslinking and ammonium ion electrostatic interaction. Three different formulations were designed to evaluate the influence of the different functional groups: the first one using CHI, the second one using carboxymethyl CHI, and the third one using a CHI-based hydrogel modified with succinic anhydride. Interestingly, the first two samples showed significant swelling at pH 1.2, probably due to the change of -NH_2_ groups to -NH_3_^+^ at an acidic pH, while the third sample’s swelling was registered at pH 7.4, attributed to the large intestine medium. Over 90% of drug loading was obtained for the different hydrogel compositions, confirming their potential use for systemic or oral drug delivery [47].

#### 5.2.2. Alginate

Alginate (ALG), or alginic acid, is one of the most commonly used polysaccharides for biomedical applications, due to its biocompatibility, low cost, low cytotoxicity, and environmental sustainability. Typically extracted from brown algae such as Laminaria hyperborean and lessonia [70], ALG is composed of homopolymeric blocks of (1,4)-linked *β*-D-mannuronic acid and *α*-L-guluronic acid bearing free functional hydroxyl and carboxyl groups and arranged in diaxial links [71]. The ratio between the structural monomers, as well as the molecular weight of polymer chains, depend on the algae of origin [72]. ALG has very low solubility in water, while it creates a viscous aqueous solution by forming a salt with monovalent cations such as sodium and potassium [73]. By changing several parameters, such as the source, the molecular weight, the chemical composition, or the density of crosslinking, it is possible to control ALG’s mechanical properties. High-molecular-weight ALG, for example, forms very viscous solutions that can affect the physical properties of the gel and can represent a risk when used with proteins or cells. Thus, it is essential to identify the appropriate combination of high- and low-molecular-weight ALG polymers to obtain the desired properties [74].

Because of the numerous hydroxyl and carboxyl groups in its backbone structure, ALG can be subjected to further chemical modifications such as crosslinking reactions. CaCl_2_ is commonly used as a crosslinking agent in ALG-based hydrogel synthesis [75] but it should be noted that it is also responsible for uncontrolled ALG gelation. The rate of gelation is, indeed, an important parameter that can affect the gelation process. A slow gelation rate results in a more uniform structure and better mechanical integrity, while a faster gelation rate may be critical for certain applications, forming heterogeneous gels with inconsistent mechanical properties [76]. Kuo et al. developed a calcium sulfate (CaSO_4_) and calcium carbonate (CaCO_3_) gelation system able to prolong the gel formation process by allowing the uniform distribution of calcium in the ALG solution before gelation occurs. This system gives the opportunity to control the homogeneity and mechanical properties of the gel by varying the total calcium content and the CaSO_4_:CaCO_3_ ratio [77].

ALG can play an important role in drug delivery applications due to its biocompatible and highly hydrophilic nature. Its use has been investigated for the delivery of a variety of compounds, such as small chemical drugs [78], proteins [79], or essential oils [80]. The high water content, the porosity, and the facile crosslinking process of ALG hydrogels make them ideal candidates as delivery systems, allowing the substantial encapsulation of the different compounds. Successful fabrication of macro-scale nitric oxide-releasing ALG beads has been accomplished by Bright et al., via the external crosslinking of the guluronate blocks of the polymer by divalent cations (Ca^2+^) on the surface. The encapsulation of S-nitrosoglutathione into ALG hydrogel beads showed promising antimicrobial activity for biomedical applications, without eliciting any cytotoxic effect [48]. However, a major drawback in ALG hydrogels is the rapid release of loaded drugs, which will diffuse without control out of the matrix, leading to an initial burst release. The synthesis of “smart” delivery systems able to release the loaded drug only if triggered by internal or external stimuli can overcome this challenge [42]. Roquero et al. realized an interpenetrating hydrogel composed of a mixture of ALG and low-molecular-weight polyvinyl alcohol (PVA) with calcium chloride and 1,3-benzenediboronic acid, which were able to efficiently seal the larger pores, slowing down by 20–30 folds the release of encapsulated proteins [49]. ALG hydrogels are also used as a matrix to immobilize nano-size species, resulting in a new class of heterogeneous materials with the properties of both components [81]. An emulsion-filled ALG hydrogel was developed as an intestinal targeting vehicle able to prolong the intestinal residence time of a second-generation iodinated contrast agent, Iohexol (IOH), and enhance the detection efficiency of intestinal diseases. Here, an ALG matrix was used to immobilize a double emulsion (W_1_/O/W_2_) and the encapsulation efficiency (EE) of IOH increased with the concentration of ALG and CaCl_2_. ALG acted as a double protecting layer able to increase the sensitivity of this system to the gastrointestinal pH and, at the same time, to slow down the release of IOH in the intestine [50]. A smart magnetic hydrogel was synthesized for the delivery of exosomes isolated from adipose-derived mesenchymal stem cells (MSCs) for more targeted delivery for tissue repair and regeneration. Magnetic materials can be incorporated into scaffolds to promote the osteogenic differentiation of stem cells and increase in vivo bone regeneration. Here, an ALG matrix containing cobalt ferrite and exosomes was developed and the combined synergy of the three elements resulted in a higher osteogenic response [51]. Polysaccharide nanocrystals have also been incorporated into ALG microcapsules to improve the stability of the hydrogel structure and modulate the release profile of an active compound. Petrova et al. used partially deacetylated chitin nanowhiskers as nanofillers for an ALG hydrogel. The positively charged amino groups of chitin were able to interact with the negatively charged carboxyl groups of ALG, forming a polyelectrolyte complex and altering the stability of the system and its mechanical properties [82]. The hydrogel was used for the vaginal delivery of metronidazole (MET), an antiprotozoal and antimicrobial agent used for the treatment of bacterial vaginosis. The presence of chitin nanowhiskers prolonged the release profile of the drug, compared to the chitin-free system, and ALG’s pH sensitivity allowed for higher MET release (65–67% for 24 h) at a target vaginal pH of 4.5. Moreover, chitin and ALG both present mucoadhesive properties, providing the system with the ability to adhere to the vaginal mucosa, increasing the drug residence time and bioavailability [52].

#### 5.2.3. Hyaluronic Acid

Hyaluronic acid (HA) is a linear, nonsulfated glucosaminoglycan and an essential component of the extracellular matrix (ECM). It consists of alternating units of a repeating disaccharide, *β*-1,4-linked glucuronic acid–*β*-1,3-linked N-acetyl-D-glucosamine. It can contain 2000 to 25,000 repeating disaccharide units, with a molecular weight ranging from 100,000 Da in serum to 8,000,000 Da in the vitreous of the eye [83]. HA plays an important role in several cellular responses, such as proliferation, differentiation, adhesion, and gene expression [84]. HA is synthesized at the inner face of the plasma membrane as a free linear polymer and translocated in the extracellular matrix by HA synthases (HASs). There are three HAS isoforms, HAS1, HAS2, and HAS3, which synthesize HA at different catalytic rates, resulting in different-sized polymers. Overexpression of these enzymes in human tumor cell lines has been correlated with increased cell proliferation and mobility [85]. To improve the biomechanical properties of HA, the polysaccharide can be modified to provide a mechanically and chemically robust material. These modifications commonly target three functional groups: the carboxylic acid, the hydroxyl functionality, and the N-acetyl group (following deamination) [86].

The most frequently used functional groups for reaction with other natural or synthetic polymers are the carboxylic acid and hydroxyl groups: the former usually undergo carbodiimide-mediated reactions, esterification, and amidation, while the latter can be modified by etherification, divinylsulfone crosslinking, esterification, and bis-epoxide crosslinking [87]. These HA derivatives can be grouped into two major categories: “monolithic” when they cannot form new chemical bonds in the presence of cells or tissues, resulting in a terminal modification that can only be processed into different physical forms, or “living” when the modification still allows the formation of new covalent bonds in the presence of cells or tissues, and they are usually employed for in vivo or in vitro biological use [88].

In the past decade, HA and its derivatives have found use in different biomedical applications because of their hydrophilicity, biocompatibility, and viscoelastic properties, and they are considered important components of synthetic scaffolds for the delivery of drugs and genes [89]. In this context, He et al. recently designed a tissue-adhesive hydrogel depot able to achieve anti-inflammatory and anti-oxidative effects [53]. The matrix consisted of methacrylic acid and 3-aminophenylboronic acid grafted to HA. Epigallocatechin-3-gallate (EGCG) was used as a crosslinking agent and bioactive drug, being able to scavenge ROS and inhibit pro-inflammatory mediators’ expression. Gelatin (Gel) was then coupled to HA to improve cells’ attachment, growth, and proliferation [90]. Photoinitiation radical polymerization improved the mechanical properties of the HA/Gel hydrogel and drastically reduced the burst release of EGCG. The pH responsiveness due to phenylboronic acid ester formation was evaluated, showing a higher cumulative release amount of EGCG at an acidic pH compared to a neutral pH, allowing us to consider the designed matrix as a possible treatment for oxidative stress and inflammation [53].

Multidrug resistance and low tumor selectivity with consequent side effects limit the use of several drugs [91]. In order to overcome these shortcomings, a pH and enzyme dual-responsive HA-based hydrogel was developed to simultaneously deliver Doxorubicin (Dox) and core–shell SiO_2_ nanoparticles containing glucose oxidase (GOD). HA was first modified with a carbon double bond and an amino group via methyl acrylate/ethylenediamine reaction and then crosslinked by diglycidyl ether (EO-PEG-EO). The modification was confirmed by Fourier transform infrared (FTIR). The hydrogel was able to release Dox by the action of esterase, inducing the apoptosis of tumor cells and GOD-loaded SiO_2_ nanoparticles at a low pH, increasing the glucose consumption and generating H_2_O_2_ by the catalysis of GOD. The in vitro and in vivo analysis revealed that the developed system represents a promising strategy to overcome chemoresistance in cancer therapy [54].

#### 5.2.4. Cellulose

Cellulose (CLS)-based hydrogels represent porous scaffolds able to imitate the roles of the extracellular matrix, finding application in different biomedical fields, such as targeted drug delivery, tissue engineering, wound dressings, and bioimaging [92]. CLS is the most abundant biopolymer and the main component of several natural fibers. It is biodegradable by nature and presents high mechanical and thermal stability, in addition to non-toxicity and a low cost [93]. It consists of repeating units of anhydro-D-glucose linked together via a *β*-(1,4)-glycosidic bond, with a chemically reducing functionality at one end and a non-reducing end with a hydroxyl group on the other side [94]. CLS has poor solubility in water, but it is possible to overcome this drawback by following several chemical modification procedures, such as esterification, etherification, and oxidation [95]. The most commonly used CLS ethers are ethyl cellulose (ET), hydroxyethyl cellulose (HEC), methylcellulose (MC), CMC, hydroxypropyl cellulose (HPC), and HPMC.

CMC is one of the most important polysaccharides, obtained by the reaction of CLS with sodium hydroxide and monochloroacetic acid, and it is available with different molecular weights, according to the degree of substitution of carboxymethyl groups per anhydroglucose unit. CMC has good stability over a wide range of pH, ranging from 3.5 to 10, and it is tasteless and without odor [96]. Recently, it has been used by Nabipour et al. for site-specific baclofen oral delivery in combination with a copper metal–organic framework, HKUST-1. Baclofen was first loaded in HKUNST-1 and, then, coated with CMC through the carboxylic acid groups. The experiments were carried out using Baclofen-HKUNST-1 and CMC/Baclofen-HKUNST-1 and they showed that the release of Baclofen from HKUNST-1 can be managed using the CMC coating and that long-term drug dosing stability was offered by CMC’s sensitivity to pH values [55].

HEC is a hydroxyethyl ether of CLS, obtained by the reaction of CLS with sodium hydroxide first and then with ethylene oxide. It is easily soluble in cold and hot water, giving transparent solutions with different viscosities according to the polymer’s concentration, type, and temperature [97]. As with CMC, HEC is an odorless, tasteless, low-cost, biodegradable, and biocompatible polymer and it finds application in several fields, such as the pharmaceutical industry, as a drug carrier, in cosmetics, and in wastewater treatment [98].

HPMC is a water-soluble CLS ether with hydration and gel-forming properties and it is a non-charged polymer able to avoid unwanted electrostatic interactions with polyelectrolyte drugs or proteins [99]. It is one of the most important hydrophilic carrier materials and it has been largely used in the preparation of various pharmaceutical formulations: Albiero et al. developed a wet sol–gel silica-based HPMC–glycerol hydrogel for protein serpin B3 (SB3) release for treating wounds [56]; Biswas et al. synthesized a super-porous hydrogel using two grades of HPMC along with Carbopol 971p for Atenolol’s sustained release in the gastrointestinal environment [57]; a hydrogel delivery system able to release an antimalaria drug, Primaquine, was studied by Permana et al. using HPMC for its thermoresponsive and mucoadhesive characteristics [58].

Polymers formed by esterification of the hydroxyl groups with various organic acids in the presence of a strong acid as a catalyst represent the second major group of CLS derivatives [100]. This type of reaction is characterized by a specific parameter, such as the degree of substitution, which denotes the number of hydroxyl groups modified per glucosydic unit and, thus, covering a range from 0 to 3 [101]. Cellulose acetate, cellulose nitrate, cellulose acetate phthalate, hydroxypropyl methylcellulose phthalate, and hydroxypropyl methylcellulose acetate succinate are the most used esters in the pharmaceutical domain. These esters are insoluble in water, pH-insensitive, and present good mechanical strength and ease of accessibility [102]. These compounds are usually resistant to acidic environments such as that of the stomach and are, therefore, suitable for enteric coatings for capsules or tablets [103].

#### 5.2.5. Carrageenan

Marine algae contain large amounts of polysaccharides, many of which are cell wall structural, but also mucopolysaccharides and storage polysaccharides. Green algae contain sulfuric acid polysaccharides, sulfated galactans, and xylans; brown algae contain ALG, fucoidan, and sargassan; red algae contain agars, porphyran as mucopolysaccharides in the intercellular spaces, starch, and carrageenan (CRG) [104]. The latter represents a group of anionic sulfated polysaccharides formed by repeating units of D-galactose and 3,6-anhydro-galactose joined by alternating *α*-(1→3) and *β*-(1→4) linkages [105]. Three are the major classes of CRGs: kappa (*κ*)-CRG, Iota (ι)-CRG, and Lambda (*λ*)-CRG, distinguished by the presence of one, two, or three ester–sulfate groups per disaccharide unit, respectively [106]. The number and position of sulfated groups influence the properties of CRG, since the higher the number of these groups, the lower the solubility temperature and the gel strength will be. *κ*-CRGs have ester sulfate content of around 25/30%. ι-CRGs have an additional sulfated group, resulting in ester sulfate content of around 28/30%. *λ*-CRGs have three sulfated groups and a total ester sulfate content of around 32/39% [107]. The ester sulfate groups are strongly anionic, similar to naturally occurring glycosaminoglycans, and are responsible for the chemical reactivity of this class of polysaccharides. They are usually neutralized by cations, such as Na^+^, K^+^, and Ca^2+^, which also determine the physical properties and the final sol–gel transition of the CRGs [108]. *κ*- and ι-CRG, in the presence of appropriate cations, form thermotropic and ionotropic hydrogels via the crosslinking of adjacent sulfate groups. *λ*-CRG’s sulfate groups are unable to crosslink and form gels and are mostly used as thickener agents [109]. The CRG type, molecular weight, concentration, and temperature determine the gel’s viscosity, which increases exponentially with the increase in the concentration, due to a higher interaction between the macromolecular chains, and decreases with an increase in temperature [110].

All these properties, together with the biocompatibility of these compounds, make CRG an ideal polymer for controlled drug delivery applications. Among the several types of CRG, *κ*-CRG is the one that draws the most attention because of its high gelling capacity. Freeze-dried hydrogels, better known as cryogels, have attracted a lot of interest in biomedical and pharmaceutical applications [39]. Recently, a CRG-based hydrogel was developed to enable the transdermal delivery of Metformin and its release behavior under an electric field. The *κ*-CRG hydrogel was synthesized via a sol–gel transition gelation process: *κ*-CRG, at different concentrations, was first dissolved in DI water at room temperature, and then the solutions were heated to 90 °C, causing the breakdown of the *κ*-CRG chains. During the final cooldown, the gelation process occurred and the *κ*-CRG chains formed a double helix structure and, then, an aggregated double-helix structure that could be reversed again to a random-coil structure by increasing the temperature. Finally, the obtained hydrogels were frozen and dried using a freeze-drying technique to obtain the porous cryogels. The material was characterized in terms of pore size and shape: higher *κ*-CRG concentrations resulted in smaller pore sizes. With increasing pore size, the diffusion coefficient for Metformin slightly increased and the application of a positive electrical potential shorted the time of equilibrium of the drug, enhancing the release rate from the matrix. By combining *κ*-CRG’s properties and electrical potential, it was then possible to increase and control the transdermal release of Metformin [59].

While *κ*-CRG is able to form a strong gel in the presence of specific monovalent cations, ι-CRG is more sensitive to divalent ions and forms a soft gel [111]. Kochkina et al. realized a ι-CRG-based hydrogel containing Methotrexate (MTX), a strong therapeutic agent for various types of diseases. ι-CRG was dissolved in water and the solution was heated to 45 °C and then cooled at room temperature [60]. Because of MTX’s poor solubility in water, *β*-cyclodextrins (*β*CDs) were added as additives, displaying solubilizing abilities [112] and increasing the MTX content in the hydrogel. MTX and *β*CDs did not affect ι-CRG’s viscoelastic properties and, thus, the formation of the gel. The obtained release study’s results showed that the ι-CRG-MTX-*β*CD system is able to reduce MTX’s permeation and to prolong MTX’s transmembrane transport [60].

## *6.* Drug Delivery in Cancer Therapy

Cancer is the leading cause of death and an important barrier to increased life expectancy in every country in the world [113]. Current strategies, such as chemotherapy, surgery, and radiotherapy, are widely adopted for cancer treatment. In particular, chemotherapy represents the standard cancer therapy and is known to be effective in the treatment of several types of tumors, while it does not have curative effects on other types of cancer. Drawbacks related to chemotherapy lie in the numerous side effects, which can be mild, moderate, or severe according to the intensity of the treatment [114]. The main problem at present is represented by the lack of specificity of many antitumor drugs, which are not able to cause the selective death of tumor cells. The use of delivery systems to control the release of chemotherapeutics allows us to avoid some disadvantages of conventional therapies. Polysaccharide-based hydrogels’ application has drawn increasing attention in cancer treatment research because of their easy and low-cost production, biocompatibility, degradability, and non-toxicity [115]. The presence of multi-functional groups in their backbone, such as hydroxyls, amines, and carboxyls, permits easy chemical modifications to obtain polysaccharide derivatives with unique properties for specific applications. Moreover, several polysaccharides have the unique, innate ability to recognize specific receptors overexpressed on the surfaces of diseased cells, enabling the design of targeted DDS that can selectively deliver therapeutic agents through receptor-mediated endocytosis [116]. Below, we present recent advances in drug delivery system applications for different types of tumors (Table 3).

### 6.1. Breast Cancer

The clinical and molecular heterogeneity of breast cancer is well known. Worldwide, it is emerging as the leading cancer type, threatening human health, and has a mortality-to-incidence ratio of 15%. There is an urgent need to identify and improve systemic treatments that specifically target tumor cells [128]. In this regard, Ma et al. used microfluidic electrospraying for the synthesis of CMC-based hydrogel microparticles for the efficient and specific local delivery of DOX. CMC’s highly active hydroxyl and carboxyl groups allowed its effective crosslinking by multivalent metal cations, FeCl_3_, to generate hydrogels [129]. Drug-loaded microparticles were then formed by soaking CMC hydrogels in a DOX solution and subsequent freeze-drying. Hydrogels’ biocompatibility was evaluated on murine breast cancer 4T1 and human breast cancer MDA-MB-231 cells, showing no cytotoxic effect and confirming the cytocompatible and non-toxic nature of CMC. Free DOX and CMC–DOX microparticles’ activity was then compared in a 4T1 tumor-bearing mouse model. The first treatment caused some systemic toxicity, which was negligible in the second group, due to sustained DOX release. Interestingly, the CMC-based delivery system showed biocompatibility properties and lower systemic toxicity, thus representing a potential therapeutic approach for cancer treatment [118].

### 6.2. Melanoma

Skin cancer is a global public health challenge and its mortality rate continues to increase in several regions of the world. Melanoma only represents 2.3% of all skin cancers, but it is the most aggressive form, responsible for over 75% of skin cancer-related deaths [130]. Recently, an yttrium (Yb)-loaded CHI hydrogel was developed to selectively induce cell death in B-16 mouse melanoma cells (Yb). As a matter of fact, lanthanides have been widely used for cancer treatment in several types of tumors [131]. CHI and Yb^3+^ were mixed to form a composite hydrogel. The in vitro and in vivo release studies showed the inhibition of melanoma growth, induced by Yb^3+^ ions, without causing any harmful effects on skin union and peripheral normal tissue damage [123].

### 6.3. Colorectal Cancer

Colorectal cancer comprises colon and/or rectum cancer and is the second most deadly type of cancer. Its global incidence is becoming constantly higher, and it is estimated to reach more than double by 2035, especially in less developed nations, where early diagnosis and treatment are rarely available [132]. The design of novel therapeutic approaches for targeting the colorectal region is a high priority. A noteworthy example has been reported by Sheng et al. In this study, an ALG/CMC hydrogel crosslinked with CaCl_2_ was developed as a dual drug delivery system for Methotrexate and aspirin, providing both chemotherapy and pain relief to cancer patients [120]. CaCO_3_, a naturally non-toxic inorganic biomineral successfully used as a carrier for the delivery of drugs, genes, and proteins [133], was added during hydrogel preparation to improve the mechanical performance of the matrix. The addition of CMC considerably increased aspirin’s entrapment efficiency compared to ALG alone, and the combination of the two polysaccharides avoided MTX’s absorption in the stomach and small intestine simulated fluid, showing the ability of the DDS to release both drugs at appropriate organs with a specific pH [120].

### 6.4. Renal Cell Carcinoma

Renal cell carcinoma (RCC) consists of a group of cancers originating from renal tubular epithelial cells, such as clear cell RCC, papillary RCC, and chromophobe RCC, and accounts for >85% of cancers of the kidney [134]. Risk factors for RCC include obesity, hypertension, and cigarette smoking, as well as medical conditions and genetic factors [135]. Sunitinib, a multi-targeted tyrosine kinase inhibitor (TKI), has been investigated in metastatic renal cell carcinoma. Several Sunitinib-loaded hydrogels have been synthesized, from both synthetic [136] and natural polymers. Recently, Jafari et al. developed a promising Sunitinib-carrying hydrogel using a mixture of *κ*-CRG and CHI, in the presence of magnetic montmorillonite. Clay was added to the CHI solution to improve the mechanical strength, and *κ*-CRG was used for its anionic sulfate groups, able to crosslink to amines on CHI. This system enabled the release of the drug, with an increase at an acidic pH, typical of damaged cancerous tissues [127].

## 7. Conclusions and Future Perspectives

In this review, we provided an overview on the development of hydrogels based on different polysaccharides with special focus on drug delivery applications. We classified the hydrogels on different bases, such as physical and chemical crosslinking, natural or synthetic sources, and polymeric composition. The main properties of hydrogels were described: biocompatibility and biodegradability represent two of the most important features that make polysaccharides an excellent alternative to synthetic monomers for biological applications. Different procedures can be used for the synthesis of hydrogels, including several chemical crosslinking strategies and physical stimuli. The impact of polysaccharide-based hydrogels for drug delivery is increasing day by day. In vitro and in vivo experiments demonstrated the successful application of these materials, confirming the ability of carbohydrate polymers to deliver and release therapeutics, minimizing drug loss and side effects. Anticancer drugs have been successfully delivered to targeted tumor tissue, enhancing tumor cell death and reducing side effects on normal cells. Many natural hydrogel compositions have been tested on different types of tumors and no cytotoxic effect has been recorded due to their biocompatible and biodegradable properties. In summary, polysaccharide-based hydrogels have shown in vitro and in vivo tumoricidal activity towards cancer cells and represent promising carriers for several pharmaceutical agents to target cells.

Although we have highlighted the advantages of using natural hydrogels for controlled drug delivery, the clinical success of these systems remains limited. More comprehensive research is needed to fully understand and exploit the potential of these formulations. Unlike synthetic polymers, natural hydrogels have inferior mechanical strength. This drawback does not allow the use of hydrogels in the medical field unless they are combined with a synthetic polymer to achieve the proper reinforcement. Moreover, the necessity to improve the therapeutic effect of hydrogels still remains. The combination of different polymers, as well as the incorporation of nanoparticles or active agents in the three-dimensional network, can increase the beneficial effects of hydrogels, reducing cytotoxic reactions and obtaining the desired therapeutic activity. Many biomedical applications require variations in the dose or long-term release, normally achieved using hydrophobic systems, and, thus, it would be helpful to be able to actually control the release. For example, hydrogels with different degradation times can represent a potential strategy to modulate drug delivery over time.

The improvement of all or some of these parameters would help to fully exploit hydrogels’ abilities to deliver new drugs at specific times and locations in the body, resulting in better and less risky therapeutic procedures, especially in the treatment of cancer. Moreover, the incorporation of a contrast agent in the hydrogel structure will allow theranostic carriers to be developed for simultaneous imaging and drug release, providing better prognosis and improved treatments for cancer patients. In conclusion, we have detailed several polysaccharide-based hydrogels and their application as drug delivery systems for treating diseases. Thanks to their biocompatibility, biodegradability, low toxicity, and low cost, polysaccharides show great potential in the biomedical field, and the development of new, marketable products to treat a broad range of diseases is likely to be achieved.

## Figures and Tables

**Table 1:** Hydrogels’ classification and main features based on different parameters.

Criteria	Classification	Features
Crosslinking	Chemical	Polymers are covalently crosslinked by permanent junctions. It can be carried out by the addition of crosslinker molecules, polymer–polymer conjugation, or photoinitiators.
	Physical	Polymers are hold together by chain entanglements and/or hydrogen bonds or hydrophobic or ionic interactions.
Polymeric composition	Homopolymer	Hydrogel derived from a single species of monomer.
	Copolymer	Hydrogel consists of two or more different monomers with at least one hydrophilic component.
	Semi-interpenetrating network	Hydrogel consists of one crosslinked monomer and another non-crosslinked component.
	Interpenetrating network	Hydrogel are made of two independent crosslinked polymeric chains contained in a network form.
Source	Natural	Polysaccharides and proteins are examples of polymers for natural hydrogels. They are biocompatible and biodegradable.
	Synthetic	Synthetic hydrogels have higher strength and can be designed to have specific mechanical and chemical properties.
	Hybrid	Hydrogels consists of a combination of synthetic and natural polymers.
Physical structure	Amorphous	They contain randomly arranged macromolecular chains.
	Crystalline	They possess dense regions of ordered macromolecular chains.
	Semicrystalline	A mixture of amorphous and crystalline phases.
Network electrical charge	Nonionic	They do not present any charged groups and have ultra-durable and permanent connections.
	Ionic	They can be positive or negative and have different behaviors according to the pH.
	Amphoteric	Hydrogels contain both acidic and basic groups.
	Zwitterionic	They present an equal amount of positive and negative charge.

**Table 2:** Examples of natural polymer-based drug delivery systems.

Hydrogel Source	Additional Components	Synthesis Method	Loaded Drug	Reference
Chitosan	---	Formaldehyde crosslinking	DOX/5-FU	[47]
Sodium alginate	---	Ionic crosslinking	S-nitrosoglutathione	[48]
Sodium alginate	Polyvinyl alcohol/ benzeneboronic acid	Ionic crosslinking	Proteins	[49]
Sodium alginate	---	Ionic crosslinking	Iohexol	[50]
Sodium alginate	Polyvinyl pyrrolidone	Ionic crosslinking	Exosomes	[51]
Sodium alginate	Chitin nanowhiskers	Ionic crosslinking	Metronidazole	[52]
Hyaluronic acid	Gelatin	UV radiation	Epigallocatechin- 3-gallate	[53]
Hyaluronic acid	Core–shell SiO_2_ nanoparticles	UV radiation	Doxorubicin/ glucose oxidase	[54]
CMC	HKUST-1	Ionic crosslinking	Baclofen	[55]
HPMC	SiO_2_/Glycerol	Chemical crosslinking	Serpin B3	[56]
HPMC	Carbopol 971p	Chemical crosslinking	Atenolol	[57]
HPMC	Pluronic F127 (PF127) and F68 (PF68)	Temperature-dependent gelation	Primaquine	[58]
κ-carrageenan	---	Freeze-drying process	Metformin	[59]
ι-carrageenan	β-cyclodextrins	Maturation	Methotrexate	[60]

**Table 3:** Polysaccharide-based hydrogels and their latest applications as drug delivery systems in different types of cancer.

Cancer Type	Hydrogel Origin	Loaded Drug	In Vitro/In Vivo Outcomes	
Breast cancer	Hyaluronic acid	Doxorubicin	HA scaffold had great antitumor activity when combined with near-infrared light, showing synergistic antitumor and photothermal effect.	[117]
Breast cancer	CMC	Doxorubicin	The system showed tumor inhibition effect with a strong apoptotic signal and no significant changes in bodyweight.	[118]
Glioblastoma	Cellulose/chitosan	TRAIL	Hydrogel scaffolds maintained cell viability and released TRAIL at concentrations that exhibited in vitro efficient tumor cell killing.	[119]
Colorectal cancer	CMC/alginate	Methotrexate/aspirin	The system showed concentration-dependent cytotoxicity with a colon cancer cell viability decrease of up to 10%.	[120]
Colorectal cancer	Chitosan/chondroitin sulfate	Curcumin	Hydrogel scaffold did not present significant level of cytotoxicity and allowed efficient drug release and absorption preferentially by cancer cells.	[121]
Lung cancer	Acylhydrazide-functionalized CMC	Limonin	Limonin-loaded hydrogels exhibited enhanced tumor suppression efficiency through a sustained release process with no difference in tissue morphology.	[122]
Melanoma	Chitosan	Ytterbium (Yb^3+^)	Chitosan hydrogel induced in vitro melanoma cells’ anoikis and inhibited tumor growth in animal experiment.	[123]
Melanoma	HPMC/Cyclodextrins	3-O-Methylquercetin (3OMQ)	The formulation achieved complete 3OMQ release using a Franz cell model, reaching the whole skin layer.	[124]
Prostate cancer	Alginate/ cyclodextrins	Paclitaxel	The combined ALG–CD complex prevented Paclitaxel crystallization and allowed its diffusion out of the network, decreasing the metabolic activity of prostate cancer cells in a dose-dependent manner.	[125]
Hepatocellular carcinoma	N-carboxyethyl chitosan	Doxorubicin	The pH-responsive system showed good degradability properties in tumor acidic microenvironment with enhanced drug efficiency to kill tumor cells and less side effects for normal tissue.	[126]
Renal cell carcinoma	Κ-carrageenan/ chitosan	Sunitinib	In vitro release studies showed pH-dependent release of the drug, with an increase at acidic pH similar to damaged cancerous tissues.	[127]

## Data Availability

The data availability statement is not applicable to this study.
